# Multiple Ipsilateral Acute Epidural Hematomas: A Case Report and Literature Review

**DOI:** 10.7759/cureus.90256

**Published:** 2025-08-16

**Authors:** Ryosuke Dowaki, Shumpei Onishi, Hiroshi Kondo, Fumiyuki Yamasaki, Nobutaka Horie

**Affiliations:** 1 Department of Neurosurgery, Graduate School of Biomedical and Health Sciences, Hiroshima University, Hiroshima, JPN; 2 Department of Neurosurgery, Matsuyama Red Cross Hospital, Matsuyama, JPN; 3 Department of Neurosurgery, National Hospital Organization Kure Medical Center and Chugoku Cancer Center, Kure, JPN

**Keywords:** acute epidural hematoma, computed tomography angiography, contrast extravasation, head trauma, hematoma evacuation

## Abstract

Acute epidural hematoma (AEDH) typically develops from a skull fracture caused by head injuries and usually presents as a single lesion. The surgical strategy becomes more complex when multiple AEDHs are present. In this report, we present the usefulness of CT angiography for detecting active bleeding lesions in multiple AEDHs. A 33-year-old man was transferred to our hospital with a head injury. Two isolated right-sided AEDHs were observed on CT: a thick temporoparietal lesion causing a midline shift and another thin lesion in the middle cranial fossa. Contrast-enhanced CT revealed extravasation in both hematoma cavities, and CT angiography revealed pseudoaneurysms along with the middle meningeal artery at both AEDHs. The patient underwent simultaneous evacuation of both hematomas. Active bleeding was observed at the pseudoaneurysm lesions identified on the CT angiography, and the bleeding arteries had coagulated. The patient recovered without any neurological deficit. Our case clearly showed that contrast-enhanced CT and CT angiography were valuable for visualizing the active bleeding associated with AEDHs.

## Introduction

Acute epidural hematomas (AEDHs) develop between the dura mater and skull after a head injury, causing a skull fracture, typically due to disruption of the middle meningeal artery (MMA), diploic vein, or venous sinuses [1]. Although AEDHs commonly occur as solitary lesions, they rarely present as multiples, which are associated with higher mortality rates [2]. In general, urgent surgical evacuation of a hematoma is required for patients with a large hematoma (>30 cm^3^) or progressive neurological deficit [1]. Nonoperative treatment is applied to patients with small hematomas and stable neurological symptoms [3]. In patients with multiple AEDHs, choosing a therapeutic strategy might be difficult depending on the conditions of each hematoma.

In the guidelines for traumatic brain injury, non-contrast computed tomography (CT) findings and neurological symptoms are key parameters that can indicate the need for surgical treatment of AEDH [1]. The utility of contrast-enhanced CT and CT angiography is well-established for detecting active bleeding vessels and predicting hematoma expansion in intraparenchymal hemorrhage and subdural hematomas, but these techniques have rarely been used for assessing AEDH [4,5]. In this report, we present a rare case of multiple ipsilateral AEDHs and discuss the utility of contrast-enhanced CT and CT angiography in AEDH. These techniques identified the active bleeding site and helped physicians plan the optimal therapeutic strategies for multiple AEDH.

## Case presentation

A 33-year-old male, who was working with high-voltage power units, accidentally touched the electric device and got an electric shock. Subsequently, he collapsed due to the electric shock and suffered a head injury. His colleague found him, and he was transferred to the emergency department.

When he was transferred to our hospital, he was alert and could communicate with the medical staff. He had no history of disease or regular medication. On the physical examination, electrical burns were observed on his face and extremities, but vital signs were within normal range. On the neurological examination, his Glasgow Coma Scale (GCS) score was 15 without anisocoria, and he showed no motor or sensory deficit. Laboratory tests, including platelet count and coagulation profile, were within the normal range.

**Figure 1:**
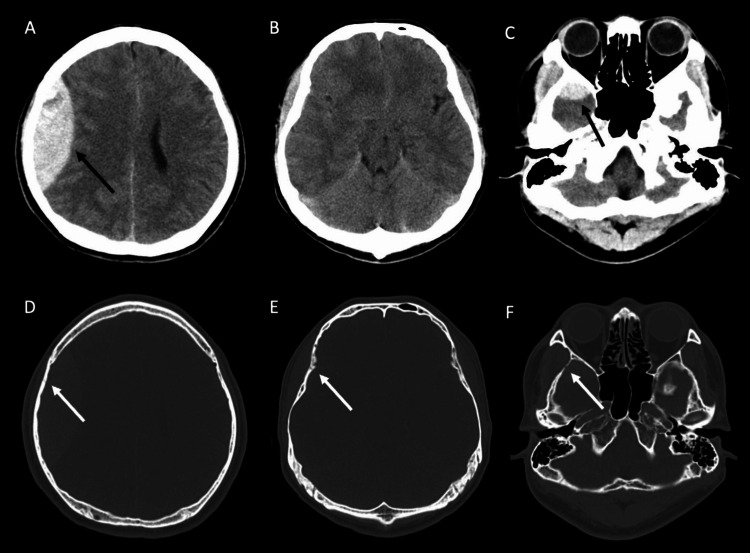
Preoperative non-contrast CT image (A) A non-contrast CT scan showed a large AEDH at the right temporoparietal region, with a volume of approximately 80 cm^3^ (black arrow). The AEDH caused a mass effect on the brain. (B) A non-contrast CT scan at the level of the midbrain did not show AEDH and other brain injuries, suggesting the AEDH at the right middle-fossa lesion and the right temporoparietal lesion are separately located. (C) A non-contrast CT scan showed a small AEDH at the right middle-cranial fossa, with a volume of less than 5 cm^3^ (black arrow). (D-F) Non-contrast CT scan with bone window showed a single straight fracture extending from the right temporal bone to the right parietal bone (white arrow). The bone fracture was adjacent to the AEDHs. AEDH: acute epidural hematoma; CT: computed tomography

**Figure 2:**
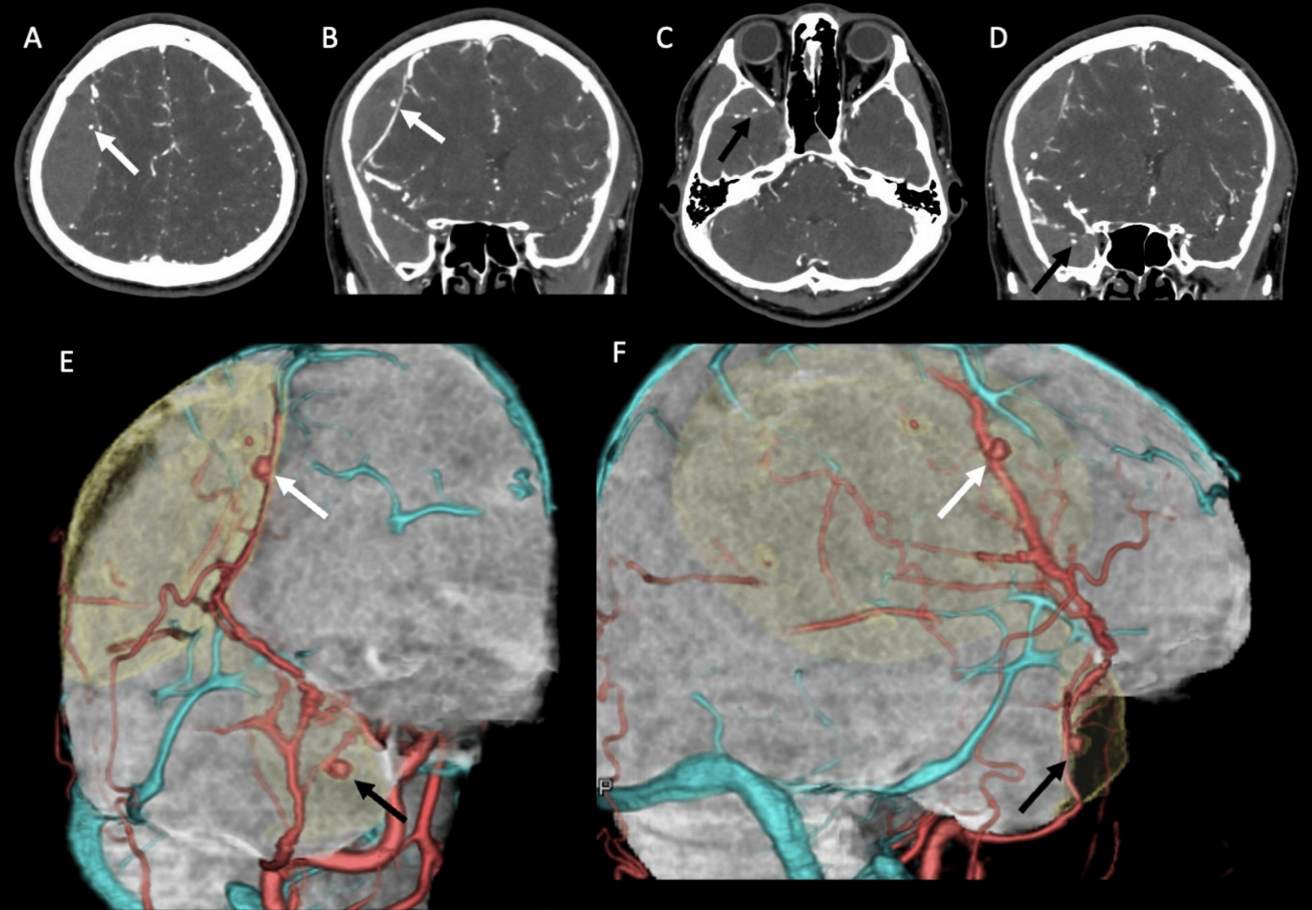
Preoperative contrast-enhanced CT image and CT angiography (A-B) Contrast-enhanced axial (A) and coronal (B) CT scans demonstrated the contrast-medium extravasation (white arrow) in the small AEDH at the right temporoparietal region. (C-D) Contrast-enhanced axial (C) and coronal (D) CT scans demonstrated the contrast-medium extravasation (black arrow) in the small AEDH at the right-middle cranial fossa. (E-F) An anterior view (E) and a right lateral view (F) of CT angiographies demonstrated two pseudoaneurysms in the right temporoparietal AEDH (white arrow) and right middle-cranial fossa AEDH (black arrow) along with the right middle meningeal artery. The AEDHs are colored in light yellow. AEDH: acute epidural hematoma; CT: computed tomography

After the general examination, he underwent a CT scan. Non-contrast CT revealed two distinct AEDHs at the right temporoparietal region and the right middle-fossa region (Figures 1A-1C). The epidural hematomas were completely separated and not connected to each other. With a bone window image of the CT scan, a single straight fracture was observed extending from the temporal bone to the parietal bone (Figures 1D-1F). These findings suggest that a single head injury caused two distinct epidural hematomas. One hematoma in the right temporoparietal region was thick and large (approximately 80 cm^3^), causing a midline shift (Figure 1A). Another smaller hematoma (<5 cm^3^) was located in the right middle-cranial fossa, which did not cause a significant mass effect (Figure 1C). There were no other obvious injuries, such as subdural hematomas or cerebral contusions. Since he suffered an electrical injury that sometimes causes vascular injuries not only in the trunk but also in the intracranial and peripheral vessels [6,7], we performed contrast-enhanced CT and CT angiography. Subsequently, we obtained a contrast-enhanced CT scan and CT angiography. Contrast-enhanced CT showed two sites of contrast-medium extravasation along with the MMA in the large temporoparietal hematoma (Figures 2A-2B) and also in the small right middle-cranial fossa hematoma (Figures 2C-2D). Additionally, CT angiography revealed pseudoaneurysms at both AEDHs (Figures 2E-2F).

**Figure 3:**
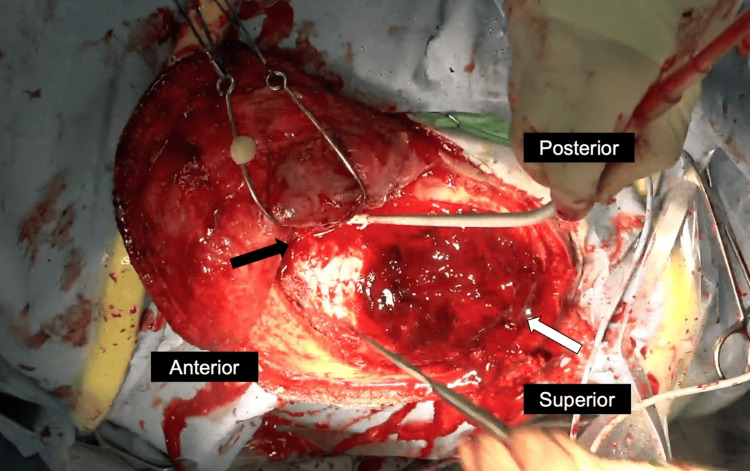
Intraoperative findings Post-craniotomy view shows two distinct AEDHs at the right middle-cranial fossa region and the right temporoparietal region. The small hematoma in the middle cranial fossa showed active bleeding from the middle meningeal artery (black arrow). A large hematoma was located at the temporoparietal region (white arrow). AEDH: acute epidural hematoma

His consciousness deteriorated to give a GCS score of 14 (E = 3, V = 5, M = 6) after the CT scan; hence, an urgent hematoma evacuation was planned. Although one of the hematomas was relatively small, the detection of extravasation and pseudoaneurysms in both hematoma cavities reinforced our decision to evacuate both hematomas simultaneously. A question mark-shaped skin incision was made from the frontal region to the area anterior to the right auricle. A single large craniotomy encompassing both hematomas was performed, and the hematomas were removed. Active arterial bleeding from the MMA was observed after removing the small AEDH at the middle-cranial fossa, consistent with the pseudo-aneurysm identified on CT angiography (Figure 3, black arrow). We coagulated the MMA, and the bleeding from pseudoaneurysms stopped. Postoperative CT showed evacuation of the hematomas. Postoperative MRI did not show any diffuse axonal injuries (DAI). The patient was discharged without any neurological deficit.

## Discussion

This rare case of multiple ipsilateral AEDHs demonstrated the utility of contrast-enhanced CT and CT angiography for detecting active hemorrhage and fragile vascular abnormalities of AEDHs.

The overall incidence of multiple AEDHs is 2%-10% among all AEDHs [8,9]. Most of the multiple AEDHs are bilateral, whereas ipsilateral cases are uncommon. A single institutional retrospective study reported that 46 out of 1,025 AEDH cases (3.8%) presented with multiple AEDHs, of which only four cases (0.4%) were ipsilateral [2]. To clarify the characteristics of multiple AEDHs, we performed a literature review and found 62 reported patients with multiple AEDHs (Table 1) [2,8-16]. Among them, only seven patients showed ipsilateral multiple AEDHs. The mortality rate of the patients with multiple AEDHs was 29% in our literature review, whereas the mortality rate of a single AEDH was 9.0% in a previous study [2]. Our literature review and previous reports indicated that patients with multiple AEDH had poorer prognoses. Focusing on the ipsilateral multiple AEDH patients, two patients (28.6%) died of AEDHs. One of them died two days after surgery due to severe DAI [8]. Although five patients (71.4%) survived after surgery, they did not show obvious brain parenchymal injuries [13]. Multiple AEDHs are generally the result of severe head injury, and the rapid acceleration and deceleration of brain tissue can cause DAI and other brain parenchymal injuries [17]. These conditions may lead to a poor prognosis for these patients. Our patient, fortunately, did not experience complications with DAI and fully recovered after surgery.

**Table 1:** A summary of the literature review of patients with multiple acute epidural hematomas. GCS: Glasgow Coma Scale

Authors	Year	Number of patients	GCS on admission (number)	Bilateral	Ipsilateral	Number of deaths
Görgülü, et al. [9]	2000	6	13-15 (0), 9-12 (3), 3-8 (3)	6	0	1
Ramzan, et al. [10]	2002	2	13-15 (2), 9-12 (0), 3-8 (0)	2	0	0
Mohanty, et al. [2]	2004	46	13-15 (9), 9-12 (16), 3-8 (21)	42	4	16
Idei, at al. [11]	2004	1	15	1	0	0
Agrawal [12]	2011	1	9	1	0	0
Udoh [16]	2011	1	11	1	0	0
Baugh, et al. [13]	2013	1	10	0	1	0
Fricia, et al. [14]	2019	1	7	1	0	0
Montemurro, et al. [8]	2020	1	7	0	1	1
Fadalla, et al. [15]	2022	1	14	1	0	0
Our case		1	15	0	1	0
Total		62	13-15 (14), 9-12 (22), 3-8 (26)	55	7	18

In addition to primary injury, preventing secondary brain injury caused by elevated intracranial pressure is important to achieve a better prognosis in patients with AEDH [18]. Hematoma evacuation is the standard treatment for the management of intracranial pressure in patients with AEDH, whereas surgical treatments would not be considered for those with small volumes of AEDH [3]. In patients with multiple AEDHs, surgical hematoma evacuation for a single lesion sometimes induces enlargement of the other untreated hematoma [14,19,20]. In patients with bilateral hematomas, estimating which hematoma carries the greater risk is important when deciding which side to operate on first. Therefore, predicting the risk of increased hematoma size is of high clinical value for appropriately managing multiple AEDHs.

Extravasation in contrast-enhanced CT reportedly is an effective predictor of hematoma expansion in intraparenchymal hemorrhage and subdural hematomas [4,5]. However, the usefulness of contrast-enhanced CT for predicting the expansion of AEDH has not yet been established. In our patient with AEDH, contrast-enhanced CT clearly revealed the extravasation in both small and large hematomas. Subsequently, CT angiography revealed pseudoaneurysms at both AEDHs, and active bleeding was confirmed at the sites of AEDHs during the surgery. Our findings in this case implied that the extravasation shown on contrast-enhanced CT could also be a predictive factor for hematoma expansion in AEDH.

Detecting active hemorrhage and fragile vascular abnormalities is valuable in unusual AEDH cases with challenging therapeutic decisions, such as small hematomas requiring risk assessment for expansion and bilateral hematomas in which the priority of surgical intervention must be decided. A previous study for small AEDH demonstrated that catheter angiography could detect active extravasation of the contrast medium and pseudoaneurysms in hematomas. The patients with small AEDH in that study were embolized, and further enlargement of the hematoma was not observed [21]. Another study compared CT angiography with catheter angiography in patients with AEDH. That study showed that CT angiography could detect the pseudoaneurysms in AEDH that were confirmed by catheter angiography [22]. Thus, CT angiography could detect fragile vascular abnormalities associated with AEDH and might help clinicians choose the surgical indication for AEDHs.

## Conclusions

This report presented a rare case of ipsilateral variously sized AEDHs. Our case showed that even a small AEDH can present active bleeding. Therefore, the surgical implications should be carefully considered regardless of the size of the AEDH. Our preoperative imaging revealed the value of contrast-enhanced CT and CT angiography for detecting active hemorrhage and fragile vascular abnormalities in AEDHs. Since active bleeding can be observed regardless of AEDH size, contrast-enhanced CT and CT angiography can provide valuable information for planning optimal therapeutic strategies for AEDH. Not all cases require contrast-enhanced CT or CT angiography in all AEDH cases, but contrast-enhanced CT or CT angiography is valuable, especially for AEDH cases that are difficult to decide the therapeutic strategies.

## References

[1] Bullock MR, Chesnut R, Ghajar J (2006). Surgical management of acute epidural hematomas. Neurosurgery.

[2] Mohanty S, Huda MF, Sharma V, Tiwari Y, Choudhary A, Singh VP (2004). Double extradural hematoma: an analysis of 46 cases. Neurol India.

[3] Offner PJ, Pham B, Hawkes A (2006). Nonoperative management of acute epidural hematomas: a "no-brainer". Am J Surg.

[4] Wada R, Aviv RI, Fox AJ, Sahlas DJ, Gladstone DJ, Tomlinson G, Symons SP (2007). CT angiography "spot sign" predicts hematoma expansion in acute intracerebral hemorrhage. Stroke.

[5] Romero JM, Kelly HR, Delgado Almandoz JE, Hernandez-Siman J, Passanese JC, Lev MH, González RG (2013). Contrast extravasation on CT angiography predicts hematoma expansion and mortality in acute traumatic subdural hemorrhage. AJNR Am J Neuroradiol.

[6] Axayacalt GA, Alejandro CE, Marcos RA, Inocencio RF, Alfredo HG (2016). Brain hemorrhage after electrical burn injury: case report and probable mechanism. Surg Neurol Int.

[7] Tanda E, Genadiev G, Zappadu S (2023). Electrocution as a cause of vascular injury: case series and literature review. Ann Vasc Surg Brief Rep Innov.

[8] Montemurro N, Santoro G, Marani W, Petrella G (2020). Posttraumatic synchronous double acute epidural hematomas: two craniotomies, single skin incision. Surg Neurol Int.

[9] Görgülü A, Cobanoglu S, Armagan S, Karabagli H, Tevrüz M (2000). Bilateral epidural hematoma. Neurosurg Rev.

[10] Ramzan A, Wani A, Malik AH, Kirmani A, Wani MA (2002). Acute bilateral extradural hematomas. Neurol India.

[11] Idei M, Shima T, Nishida M (2004). A case of bilateral epidural hematomas occurring symmetrically in the parietotemporal region due to a parietooccipital injury [Article in Japanese]. Neurol Surg.

[12] Agrawal A (2011). Bilateral symmetrical parietal extradural hematoma. J Surg Tech Case Rep.

[13] Baugh AD, Baugh RF, Atallah JN, Gaudin D, Williams M (2013). Craniofacial trauma and double epidural hematomas from horse training. Int J Surg Case Rep.

[14] Fricia M, Umana GE, Scalia G, Raudino G, Passanisi M, Spitaleri A, Cicero S (2020). Posttraumatic triple acute epidural hematomas: first report of bilateral synchronous epidural hematoma and a third delayed. World Neurosurg.

[15] Fadalla T, Jalaleldean B, Suliman M, Elsayed M, Elmahdi M, Elsalawi W (2022). Post-traumatic bilateral synchronous acute extradural hematomas: a case report and review of literature. Ann Med Surg (Lond).

[16] Udoh DO (2012). Bilateral post-traumatic acute extradural hematomas: a report of four cases and review of literature. Niger J Clin Pract.

[17] Ng SY, Lee AYW (2019). Traumatic brain injuries: pathophysiology and potential therapeutic targets. Front Cell Neurosci.

[18] Tokutomi T (2005). Pathophysiology and critical care management of traumatic brain injury. Japanese J Neurosurg.

[19] Feuerman T, Wackym PA, Gade GF, Lanman T, Becker D (1988). Intraoperative development of contralateral epidural hematoma during evacuation of traumatic extraaxial hematoma. Neurosurgery.

[20] He Q, Tao CY, Fu RH, You C (2022). Multiple different remote epidural hematomas after craniotomy: a case report. World J Clin Cases.

[21] de Andrade AF, Figueiredo EG, Caldas JG (2008). Intracranial vascular lesions associated with small epidural hematomas. Neurosurgery.

[22] Paiva WS, Andrade AF, Amorim RL (2014). Computed tomography angiography for detection of middle meningeal artery lesions associated with acute epidural hematomas. Biomed Res Int.

